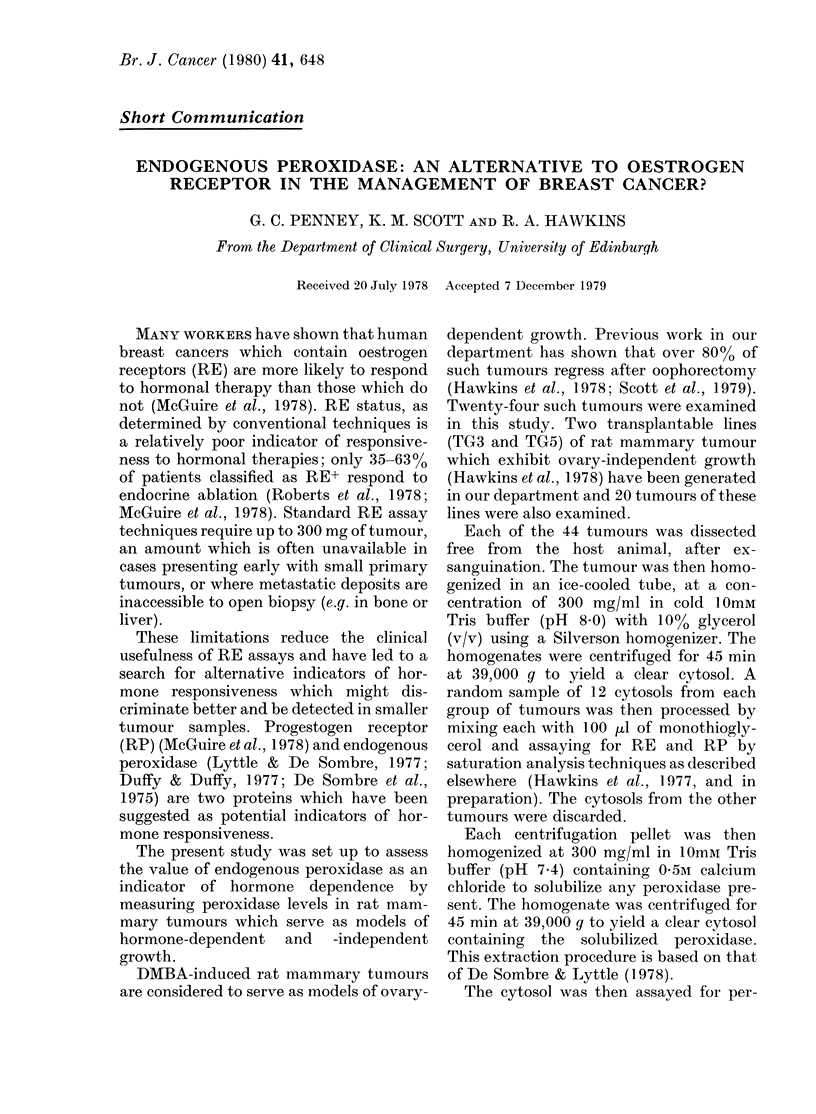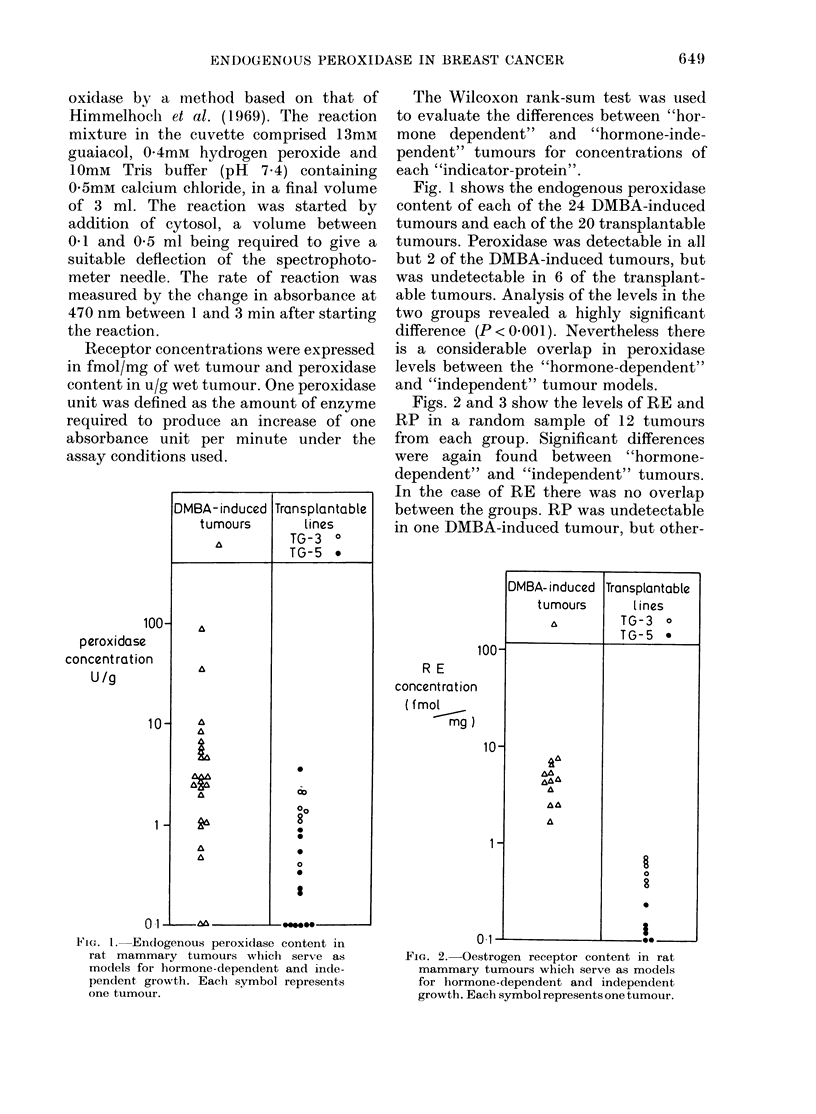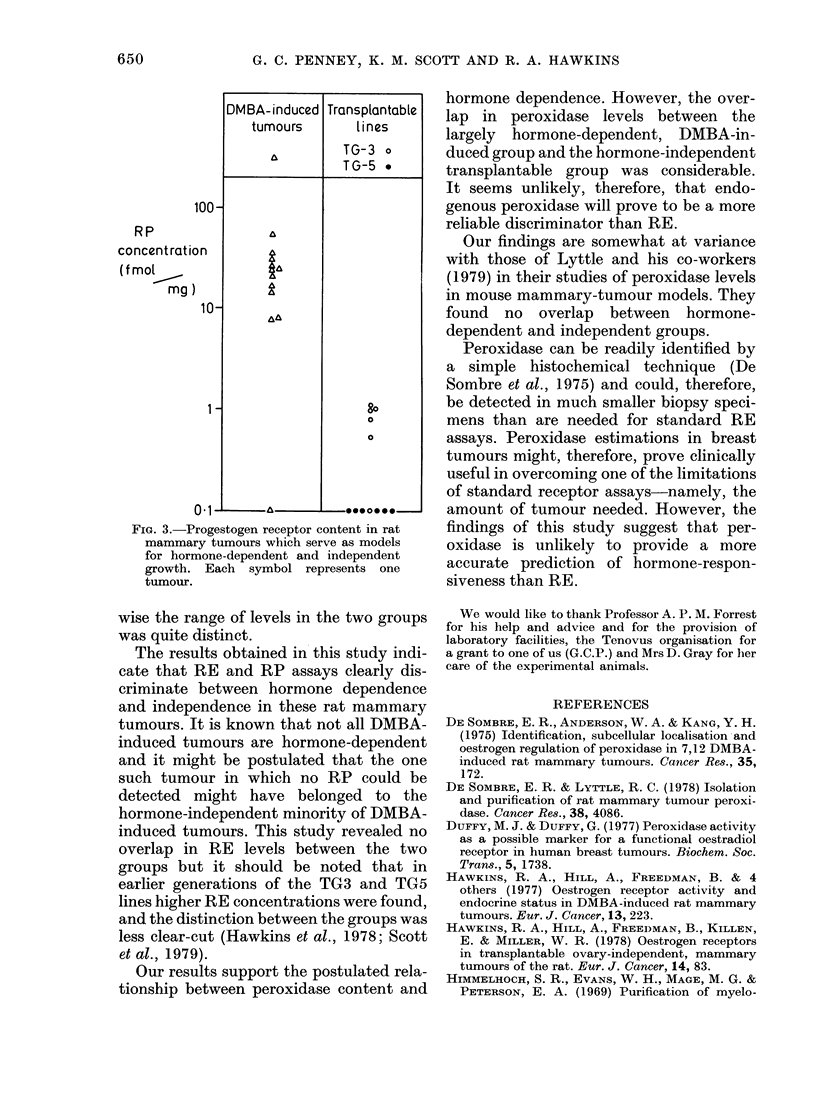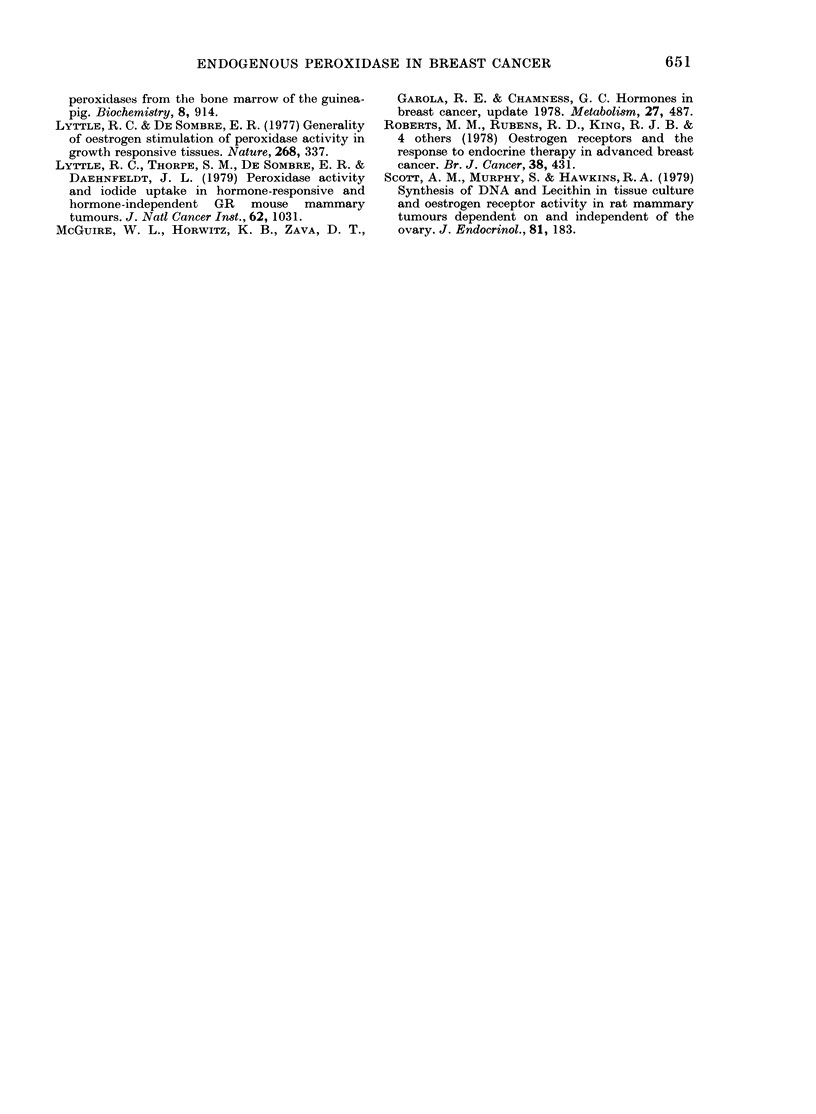# Endogenous peroxidase: an alternative to oestrogen receptor in the management of breast cancer?

**DOI:** 10.1038/bjc.1980.111

**Published:** 1980-04

**Authors:** G. C. Penney, K. M. Scott, R. A. Hawkins


*Br. J. Cancer* (1980) **41**, 648

***Short Communication***

### ENDOGENOUS PEROXIDASE: AN ALTERNATIVE TO OESTROGEN RECEPTOR IN THE MANAGEMENT OF BREAST CANCER?

G. C. PENNEY, K. M. SCOTT AND R. A. HAWKINS

*From the Department of Clinical Surgery, University of Edinburgh*

Received 20 July 1978 Accepted 7 December 1979

MANY WORKERS have shown that human breast cancers which contain oestrogen receptors (RE) are more likely to respond to hormonal therapy than those which do not (McGuire *et al.*, 1978). RE status, as determined by conventional techniques is a relatively poor indicator of responsiveness to hormonal therapies; only 35–63% of patients classified as $RE^+$ respond to endocrine ablation (Roberts *et al.*, 1978; McGuire *et al.*, 1978). Standard RE assay techniques require up to 300 mg of tumour, an amount which is often unavailable in cases presenting early with small primary tumours, or where metastatic deposits are inaccessible to open biopsy (*e.g.* in bone or liver).

These limitations reduce the clinical usefulness of RE assays and have led to a search for alternative indicators of hormone responsiveness which might discriminate better and be detected in smaller tumour samples. Progestogen receptor (RP) (McGuire *et al.*, 1978) and endogenous peroxidase (Lyttle & De Sombre, 1977; Duffy & Duffy, 1977; De Sombre *et al.*, 1975) are two proteins which have been suggested as potential indicators of hormone responsiveness.

The present study was set up to assess the value of endogenous peroxidase as an indicator of hormone dependence by measuring peroxidase levels in rat mammary tumours which serve as models of hormone-dependent and -independent growth.

DMBA-induced rat mammary tumours are considered to serve as models of ovary-dependent growth. Previous work in our department has shown that over 80% of such tumours regress after oophorectomy (Hawkins *et al.*, 1978; Scott *et al.*, 1979). Twenty-four such tumours were examined in this study. Two transplantable lines (TG3 and TG5) of rat mammary tumour which exhibit ovary-independent growth (Hawkins *et al.*, 1978) have been generated in our department and 20 tumours of these lines were also examined.

Each of the 44 tumours was dissected free from the host animal, after exsanguination. The tumour was then homogenized in an ice-cooled tube, at a concentration of 300 mg/ml in cold 10mM Tris buffer (pH 8·0) with 10% glycerol (v/v) using a Silverson homogenizer. The homogenates were centrifuged for 45 min at 39,000 *g* to yield a clear cytosol. A random sample of 12 cytosols from each group of tumours was then processed by mixing each with 100 $\mu$l of monothioglycerol and assaying for RE and RP by saturation analysis techniques as described elsewhere (Hawkins *et al.*, 1977, and in preparation). The cytosols from the other tumours were discarded.

Each centrifugation pellet was then homogenized at 300 mg/ml in 10mM Tris buffer (pH 7·4) containing 0·5M calcium chloride to solubilize any peroxidase present. The homogenate was centrifuged for 45 min at 39,000 *g* to yield a clear cytosol containing the solubilized peroxidase. This extraction procedure is based on that of De Sombre & Lyttle (1978).

The cytosol was then assayed for peroxidase by a method based on that of Himmelhoch *et al.* (1969). The reaction mixture in the cuvette comprised 13mM guaiacol, 0·4mM hydrogen peroxide and 10mM Tris buffer (pH 7·4) containing 0·5mM calcium chloride, in a final volume of 3 ml. The reaction was started by addition of cytosol, a volume between 0·1 and 0·5 ml being required to give a suitable deflection of the spectrophotometer needle. The rate of reaction was measured by the change in absorbance at 470 nm between 1 and 3 min after starting the reaction.

Receptor concentrations were expressed in fmol/mg of wet tumour and peroxidase content in u/g wet tumour. One peroxidase unit was defined as the amount of enzyme required to produce an increase of one absorbance unit per minute under the assay conditions used.

FIG. 1.—Endogenous peroxidase content in rat mammary tumours which serve as models for hormone-dependent and independent growth. Each symbol represents one tumour.

The Wilcoxon rank-sum test was used to evaluate the differences between "hormone dependent" and "hormone-independent" tumours for concentrations of each "indicator-protein".

Fig. 1 shows the endogenous peroxidase content of each of the 24 DMBA-induced tumours and each of the 20 transplantable tumours. Peroxidase was detectable in all but 2 of the DMBA-induced tumours, but was undetectable in 6 of the transplantable tumours. Analysis of the levels in the two groups revealed a highly significant difference ($P < 0{\cdot}001$). Nevertheless there is a considerable overlap in peroxidase levels between the "hormone-dependent" and "independent" tumour models.

Figs. 2 and 3 show the levels of RE and RP in a random sample of 12 tumours from each group. Significant differences were again found between "hormone-dependent" and "independent" tumours. In the case of RE there was no overlap between the groups. RP was undetectable in one DMBA-induced tumour, but other-

FIG. 2.—Oestrogen receptor content in rat mammary tumours which serve as models for hormone-dependent and independent growth. Each symbol represents one tumour.

FIG. 3.—Progestogen receptor content in rat mammary tumours which serve as models for hormone-dependent and independent growth. Each symbol represents one tumour.

wise the range of levels in the two groups was quite distinct.

The results obtained in this study indicate that RE and RP assays clearly discriminate between hormone dependence and independence in these rat mammary tumours. It is known that not all DMBA-induced tumours are hormone-dependent and it might be postulated that the one such tumour in which no RP could be detected might have belonged to the hormone-independent minority of DMBA-induced tumours. This study revealed no overlap in RE levels between the two groups but it should be noted that in earlier generations of the TG3 and TG5 lines higher RE concentrations were found, and the distinction between the groups was less clear-cut (Hawkins *et al.*, 1978; Scott *et al.*, 1979).

Our results support the postulated relationship between peroxidase content and hormone dependence. However, the overlap in peroxidase levels between the largely hormone-dependent, DMBA-induced group and the hormone-independent transplantable group was considerable. It seems unlikely, therefore, that endogenous peroxidase will prove to be a more reliable discriminator than RE.

Our findings are somewhat at variance with those of Lyttle and his co-workers (1979) in their studies of peroxidase levels in mouse mammary-tumour models. They found no overlap between hormone-dependent and independent groups.

Peroxidase can be readily identified by a simple histochemical technique (De Sombre *et al.*, 1975) and could, therefore, be detected in much smaller biopsy specimens than are needed for standard RE assays. Peroxidase estimations in breast tumours might, therefore, prove clinically useful in overcoming one of the limitations of standard receptor assays—namely, the amount of tumour needed. However, the findings of this study suggest that peroxidase is unlikely to provide a more accurate prediction of hormone-responsiveness than RE.

We would like to thank Professor A. P. M. Forrest for his help and advice and for the provision of laboratory facilities, the Tenovus organisation for a grant to one of us (G.C.P.) and Mrs D. Gray for her care of the experimental animals.

### REFERENCES

DE SOMBRE, E. R., ANDERSON, W. A. & KANG, Y. H. (1975) Identification, subcellular localisation and oestrogen regulation of peroxidase in 7,12 DMBA-induced rat mammary tumours. *Cancer Res.*, **35**, 172.

DE SOMBRE, E. R. & LYTTLE, R. C. (1978) Isolation and purification of rat mammary tumour peroxidase. *Cancer Res.*, **38**, 4086.

DUFFY, M. J. & DUFFY, G. (1977) Peroxidase activity as a possible marker for a functional oestradiol receptor in human breast tumours. *Biochem. Soc. Trans.*, **5**, 1738.

HAWKINS, R. A., HILL, A., FREEDMAN, B. & 4 others (1977) Oestrogen receptor activity and endocrine status in DMBA-induced rat mammary tumours. *Eur. J. Cancer*, **13**, 223.

HAWKINS, R. A., HILL, A., FREEDMAN, B., KILLEN, E. & MILLER, W. R. (1978) Oestrogen receptors in transplantable ovary-independent, mammary tumours of the rat. *Eur. J. Cancer*, **14**, 83.

HIMMELHOCH, S. R., EVANS, W. H., MAGE, M. G. & PETERSON, E. A. (1969) Purification of myeloperoxidases from the bone marrow of the guinea-pig. *Biochemistry*, **8**, 914.

LYTTLE, R. C. & DE SOMBRE, E. R. (1977) Generality of oestrogen stimulation of peroxidase activity in growth responsive tissues. *Nature*, **268**, 337.

LYTTLE, R. C., THORPE, S. M., DE SOMBRE, E. R. & DAEHNFELDT, J. L. (1979) Peroxidase activity and iodide uptake in hormone-responsive and hormone-independent GR mouse mammary tumours. *J. Natl Cancer Inst.*, **62**, 1031.

MCGUIRE, W. L., HORWITZ, K. B., ZAVA, D. T., GAROLA, R. E. & CHAMNESS, G. C. Hormones in breast cancer, update 1978. *Metabolism*, **27**, 487.

ROBERTS, M. M., RUBENS, R. D., KING, R. J. B. & 4 others (1978) Oestrogen receptors and the response to endocrine therapy in advanced breast cancer. *Br. J. Cancer*, **38**, 431.

SCOTT, A. M., MURPHY, S. & HAWKINS, R. A. (1979) Synthesis of DNA and Lecithin in tissue culture and oestrogen receptor activity in rat mammary tumours dependent on and independent of the ovary. *J. Endocrinol.*, **81**, 183.